# Parallel Molecular Evolution of Catalases and Superoxide Dismutases—Focus on Thermophilic Fungal Genomes

**DOI:** 10.3390/antiox9111047

**Published:** 2020-10-27

**Authors:** Katarína Chovanová, Miroslav Böhmer, Andrej Poljovka, Jaroslav Budiš, Jana Harichová, Tomáš Szemeš, Marcel Zámocký

**Affiliations:** 1 Laboratory for Phylogenomic Ecology, Institute of Molecular Biology, Slovak Academy of Sciences, Dúbravska cesta 21, SK-84551 Bratislava, Slovakia; katarina.chovanova@savba.sk (K.C.); andrej.poljovka@savba.sk (A.P.); jana.harichova@savba.sk (J.H.); 2 Department of Molecular Biology, Faculty of Nat. Sciences, Science Park of Comenius University, Comenius University, Ilkovičova 8, SK-84104 Bratislava, Slovakia; bohmer6@uniba.sk (M.B.); jaroslav.budis@uniba.sk (J.B.); tomas.szemes@uniba.sk (T.S.); 3 Department of Chemistry, Institute of Biochemistry, BOKU, University of Natural Resources and Life Sciences, Muthgasse 18, A-1190 Vienna, Austria

**Keywords:** catalase, superoxide dismutase, oxidative stress, molecular evolution, thermophilic fungi, phylogenomics

## Abstract

Catalases (CAT) and superoxide dismutases (SOD) represent two main groups of enzymatic antioxidants that are present in almost all aerobic organisms and even in certain anaerobes. They are closely interconnected in the catabolism of reactive oxygen species because one product of SOD reaction (hydrogen peroxide) is the main substrate of CAT reaction finally leading to harmless products (i.e., molecular oxygen and water). It is therefore interesting to compare the molecular evolution of corresponding gene families. We have used a phylogenomic approach to elucidate the evolutionary relationships among these two main enzymatic antioxidants with a focus on the genomes of thermophilic fungi. Distinct gene families coding for CuZnSODs, FeMnSODs, and heme catalases are very abundant in thermophilic Ascomycota. Here, the presented results demonstrate that whereas superoxide dismutase genes remained rather constant during long-term evolution, the total count of heme catalase genes was reduced in thermophilic fungi in comparison with their mesophilic counterparts. We demonstrate here, for the newly discovered ascomycetous genes coding for thermophilic superoxide dismutases and catalases (originating from our sequencing project), the expression patterns of corresponding mRNA transcripts and further analyze translated protein sequences. Our results provide important implications for the physiology of reactive oxygen species metabolism in eukaryotic cells at elevated temperatures.

## 1. Introduction

Molecular oxygen (O_2_), a product of oxygenic photosynthesis, was first released in the terrestrial biosphere in huge amounts by cyanobacteria approximately 2.32 billion years ago, and during further evolution within the Proterozoic eon, became the most abundant oxidant existing in the biosphere [1]. The aerobic metabolism that makes use of freely available O_2_ in order to produce ATP necessarily results in the generation of reactive oxygen species (ROS), viz., superoxide radical (O_2_^•−^), hydrogen peroxide (H_2_O_2_), organic—mainly lipid peroxides (ROOH), hydroxyl radical (*OH), hydroperoxyl radical (*OOH), singlet oxygen (^1^O_2_^−^), hypochlorite (OCl^−^), and nitric oxide radical (*NO). All these substances exhibit a rather high (and diverse) reactivity towards numerous cellular macromolecules, mainly including nucleic acids and proteins. At their elevated concentrations they can lead to irreversible damages and even cell death. It is therefore logical that in all aerobically metabolizing organisms (both prokaryotic and eukaryotic), a battery of antioxidants occurred to cope with such toxic and deleterious substances. Three major groups of antioxidant enzymes are represented by superoxide dismutase (SOD), catalase (CAT), and glutathione peroxidase (GPx). They evolved over long time periods in living systems to play a fundamental and indispensable role in the antioxidant protective capacity of most known biological systems mainly against free radical attacks [2]. The superoxide radical generated in various tissues through catabolic pathways is efficiently converted to hydrogen peroxide (H_2_O_2_) and molecular oxygen (O_2_) by superoxide dismutase (Equation (1)). When uncontrollably accumulated, H_2_O_2_ can be potentially harmful to body tissues or cells. In contrast, at very low concentrations it has apparently a signaling effect—as reported mainly for its production by NADPH oxidases (NOX) [3]. But hydrogen peroxide can be even more dangerous. Namely, with Fe^2+^ that can frequently occur in various cells, it is converted to deleterious hydroxyl radical (*OH) and hydroperoxyl radical (*OOH) through Fenton reactions (Equation (2a,b)). In order to prevent this phenomenon, catalase, which is in eukaryotes abundant mainly in specific organelles, peroxisomes, rapidly breaks down H_2_O_2_ into water and molecular oxygen (Equation (3)), consequently curtailing free radical-induced damage. In rare cases, when the catalase is absent, like in most natural mitochondria, the majority of the health-span functions of catalase [4] can be alternatively carried out by glutathione peroxidase [5].

2 O_2_^•−^ + 2H^+^ → H_2_O_2_ + O_2_ (1)

Fe^2+^ + H_2_O_2_ → Fe^3+^ + HO* + OH^−^ (2a)

Fe^3+^ + H_2_O_2_ → Fe^2+^ + HOO* + H^+^ (2b)

2 H_2_O_2_ → 2 H_2_O + O_2_ (3)

Fungi are among those organisms that have probably evolved the most sophisticated and efficient enzyme systems that constitute main parts of their antioxidant machinery. This is documented by the presence of several isozymes for catalase and superoxide dismutase in almost all fungal species, but their role mainly among pathogenic fungi still needs to be resolved [2]. These fungal oxidoreductases are very fast in neutralizing any molecule with the potential of developing into a free radical or any substance that can induce damage to various cell components [6]. Of particular interest, is the investigation of thermostable antioxidant enzymes originating from harsh environmental conditions that belong to a group collectively called thermozymes. These catalysts shall remain active at elevated temperatures from 60 °C up to 125 °C, thus offering obvious advantages for various biotech branches. Namely, they allow increased rates of catalyzed reactions because thermozymes have significantly higher temperature optima. They frequently work with substrates that reveal decreased viscosity at high temperatures, and they can reduce the risk of contaminations for longer processes. In contrast with their mesophilic counterparts, the structure of these heat-tolerant and heat-resistant proteins exhibits mostly compact oligomers, better accessibility to their active sites, and high specific activities at elevated temperatures [7].

It shall be noted that a real heat tolerance or even a thermophilic character is not very common among all phyla of eukaryotes. From the scope of an estimated 3.0 million fungal species existing in the nature, and around 500,000 of them already described (overview in www.mycobank.org), only about 50 species have been found to be able to grow above 50 °C [8]. These species are limited to Ascomycota and Zygomycota and no thermophilic Basidiomycota representative is found yet [9].

In this contribution, we have used a phylogenomic approach for a newly sequenced thermophilic filamentous fungus *Chaetomium thermophilum* var. *dissitum* focused on genes coding for main antioxidant enzymes superoxide dismutase and catalase to compare them with corresponding sequences from a large number of thermophilic and mesophilic organisms.

## 2. Materials and Methods

### 2.1. Fungal Material and Isolation of Genomic DNA

The strain of *Chaetomium thermophilum* var. *dissitum* (Cooney & R. Emerson) CBS 180.67 was obtained from CBS-KNAW Collection, Westerdijk Fungal Biodiversity Institute, Utrecht, The Netherlands. During all experiments, the fungus was cultivated on Malt Extract Peptone Glucose (MPG) medium (20 g Malt Extract, 1 g Peptone, 20 g Glucose/L; pH 5.4) or stored on MPG agar plates (additionally with 20 g agar/L) at 45 °C. 

Genomic DNA from 100 mg of frozen mycelium of this fungus was isolated with GeneJET Plant Genomic DNA Purification Kit (ThermoFisher Scientific, Waltham, MA, USA) according to manufacturer’s instructions. Control agarose gel electrophoresis of isolated gDNA in 0.7% TAE buffer showed acceptable high molecular weight profile (not shown). Sample with a concentration of 58.4 ng/µL and absorbance ratios A_260nm/280nm_ = 1.84, A_260nm/230nm_ = 1.90 was used for genome sequencing. The concentration of this sample was measured with Nanodrop 2000 (ThermoFisher Scientific, Waltham, MA, USA).

### 2.2. Whole Genome Sequencing and ORF Prediction

Genome sequencing of *Chaetomium thermophilum* var. *dissitum* was performed with Illumina MiSeq technology using the SPAdes 3.10.1 approach. Genome coverage of this sequencing was 16-fold. This entire genome was deposited in GenBank under accession number JAAFKN000000000 for the Bioproject PRJNA595853. For prediction of functional genes coding for antioxidant enzymes in this genome, Hidden Markov Model (HMM)-based methods, FGENESH and FGENESH+, located at www.softberry.com [10] trained for *Chaetomia* genomic sequences were used.

### 2.3. Detection of Native Expression of SOD and CAT Gene Paralogs from mRNA Libraries

The fungus *Chaetomium thermophilum* var. *dissitum* was grown in MPG medium with or without the addition of 2 mM H_2_O_2_ to cells in the exponential phase of growth. Total RNA was isolated from 30 mg of fresh or frozen mycelia obtained from such MPG medium by using RNeasy Plus Mini Kit (Qiagen, Hilden, Germany) according to the manufacturer’s instructions with a modification in homogenization step [11]. Isolated RNA was stored at −80 °C and used for complementary DNA synthesis. Complementary DNA (cDNA) was synthesized from 1–3 μL (0.5 μg) of total RNA using Maxima First Strand cDNA Synthesis Kit for RT-qPCR, with dsDNase (Thermo Fisher Scientific, Waltham, MA, USA), stored at −80 °C, and used as a template for RT-PCR.

Obtained DNA samples were used as templates in PCR for a detection and amplification of superoxide dismutase and catalase genes and for confirmation of transcription of these genes into mRNA (at the level of cDNA). PCR and RT-PCR amplifications were performed using Elizyme HS Robust DNA Polymerase (Elisabeth Pharmacon, Croydon, UK). Each PCR reaction contained DNA template of appropriate concentration, 1x HS Robust MIX, and 0.4 μM of a specific DNA primer pair. Primers for detection of superoxide dismutase and catalase genes were designed by us (all details in Table S1) by using Primer3 software [12] (http://bioinfo.ut.ee/primer3/). Reactions were amplified in a thermocycler (LabCycler, Göttingen, Germany) with the following program: initial denaturation 2 min at 95 °C; 35 cycles 15 s at 95 °C, 15 s at annealing temperature (dependent on Tm of primer sets; cf. Table S1), extension 15 s per kb at 72 °C, and last extension 5 min at 72 °C. PCR products were analyzed with electrophoresis in 0.9% agarose gel in 1× TAE buffer (40mM Tris, 20mM acetic acid, 1mM EDTA pH 8.3) and stained with GelRed Nucleic Acid Gel Stain (Biotium, Fremont, USA diluted 10,000—fold in distilled water). Relevant and correct amplicons were cloned into the pCR 2.1. TOPO vector and transformed into One Shot TOP10 chemically competent *E. coli* cells using TOPO TA Cloning Kit (Invitrogen, Carlsbad, CA, USA) according to the manufacturer’s instructions. Clones were selected on LB plates with kanamycin (50 μg/mL) or ampicillin (100 μg/mL), and selected clones were verified by barcode sequencing with M13 or specific primers. Sequencing was carried out by GATC Biotech, Constance, Germany.

### 2.4. Multiple Sequence Alignment and Phylogenetic Reconstruction

Protein sequences coding for enzymatic antioxidants were collected from RedoxiBase [13] and if not available in this database then from GenBank. Multiple sequence alignments of selected protein sequences coding for (a) Cu-Zn superoxide dismutases, (b) Fe-Mn superoxide dismutases, and (c) heme catalases were performed with the Muscle program [14] implemented in MEGA-X package [15] with maximum of alignment iterations set to 500. The evolutionary history on obtained alignment of catalases was inferred by using the maximum likelihood method and Whelan and Goldman model of amino acid substitutions as the optimal model for superoxide dismutases. Le_Gascuel model of amino acid substitutions as the best proven model was used for catalases within MEGA-X suite [15].

### 2.5. Modeling of Structures for Antioxidant Enzymes

Three-dimensional homology models for yet unknown structures of thermostable superoxide dismutases and heme catalases were obtained from Phyre-2 server [16] by employing the intensive mode with HMM and PSI-Blast for finding closest homologs with a known experimental structure. Obtained structural models were superimposed on experimental 3D structures using the program suite MultiProt (http://bioinfo3d.cs.tau.ac.il/MultiProt/) [17] and displayed with the WebLab Viewer programme (Accelrys Inc., San Diego, CA, USA).

## 3. Results and Discussion

### 3.1. Genomic Analyses and Detection of Specific mRNAs for Fungal Antioxidant-Coding Genes 

**Figure 1:**
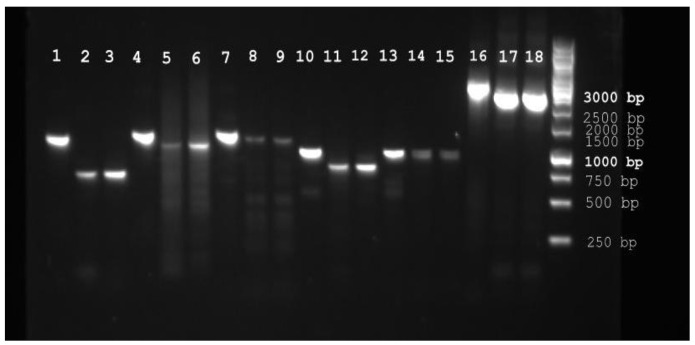
Electrophoretic profiles of obtained PCR and RT-PCR products for SOD and CAT genes from *Chaetomium thermophilum* var. *dissitum* (sequenced in this study). Agarose gel (0.9%) was run in TAE buffer (details in Section 2.3). Lane 1—genomic DNA coding for CthedisCuZnSOD1, lane 2—corresponding cDNA CthedisCuZnSOD1 control sample, lane 3—cDNA CthedisCuZnSOD1 sample induced with 2 mM H_2_O_2_, lane 4—genomic DNA of CthedisCuZnSOD2, lane 5—cDNA CthedisCuZnSOD2 control sample, lane 6—cDNA CthedisCuZnSOD2 sample induced with 2 mM H_2_O_2_, lane 7—genomic DNA of CthedisFeSOD, lane 8—cDNA CthedisFeSOD control sample, lane 9—cDNA CthedisFeSOD sample induced with 2 mM H_2_O_2_, lane 10—genomic DNA of CthedisMnSOD1, lane 11—cDNA CthedisMnSOD1 control sample, lane 12—cDNA CthedisMnSOD1 sample induced with 2 mM H_2_O_2_, lane 13—genomic DNA of CthedisMnSOD2, lane 14—cDNA CthedisMnSOD2 control sample, lane 15—cDNA CthedisMnSOD2 sample induced with 2 mM H_2_O_2_, lane 16—genomic DNA of CthedisCAT2, lane 17—cDNA CthedisCAT2 control sample, and lane 18—cDNA CthedisCAT2 sample induced with 2 mM H_2_O_2_. The same volume of obtained PCR samples was loaded in slots 1–15 and 16–18. As standard GeneRuler 1 kb DNA Ladder from ThermoFisher Scientific was used with bands of defined size presented on the right.

We have reconstructed the detailed molecular evolution of two most important enzymatic antioxidants: superoxide dismutases and heme catalases within the fungal kingdom. These antioxidants are apparently interconnected in the catabolism of reactive oxygen species (cf. Equations (1) and (3) in Section 1). Our main focus was set on available sequences from thermophilic eukaryotes as they can pose unique features for various future applications. Complete genomic sequences have already been obtained for several thermophilic fungi, including *Myceliophthora thermophila*, *Thielavia terrestris*, *Thielavia heterothallica*, *Thermomyces lanuginosus*, *Thermomyces thermophilus*, *Rhizomucor miehei*, *Talaromyces cellulolyticus*, and *Malbranchea cinnamomea.* In general, they reveal a surprisingly high level of genetic diversity [8]. We now add the newly sequenced genome of *Chateomium thermophilum* var. *dissitum.* This is a thermophilic filamentous ascomycete in the order Sordariales, living in soil, dung, and compost heaps at temperatures up to 60 °C [18]. This newly sequenced genome with GenBank accession number JAAFKN000000000 has a total size of 28.10 Mb and a GC content of 53.47% (Figure S1). The genome of a different variant, namely *C. thermophilum* var. *thermophilum*, which was reported previously by Amlacher, mainly focused on the presence of genes coding for nucleoporins of high thermal stability [19] and afterwards substantially improved in annotation by Bock [20]. It is interesting to note that, with nearly the same overall size (28.32 Mb), it has a lower GC content (52.60%). However, in general, both these thermophilic genomes are significantly shorter than the genome of e.g., typical mesophilic and closely related fungus *Chaetomium cochliodes* (34.75 Mb) [21]. None of these sequencing attempts described genes coding for enzymatic antioxidants that are the focus of our current work. The most important genes that we have discovered in the novel genome of *C. thermophilum* var. *dissitum* are listed in Table 1. In the upper part are listed those genes that are essential for precise classification of here investigated fungal variant according to molecular taxonomy. Apart from the highly conserved region of 18S–5.8S–28S rDNA, also genes coding for β-tubulin and the second largest subunit of RNA polymerase II [22] can be used as typical fungal barcodes. In the middle and lower parts of this table, genes coding for antioxidant enzymes catalases, peroxidases, and superoxide dismutases are listed, and we reconstruct their molecular evolution here. Whereas all heme catalases form only one large superfamily, superoxide dismutases found in fungal genomes can be clearly divided in two independent gene families, namely Copper–zinc superoxide dismutases and Iron–manganese superoxide dismutases, respectively. The discovery of described antioxidant genes prompted us to investigate also their spliced transcription products. With specific gene primers (listed in Table S1), we have produced genomic PCR products and in parallel also RT-PCR products from synthesized cDNA to compare spliced transcripts of SOD and catalase genes with corresponding genomic regions (Figure 1).

From this output we can observe that the size of spliced mRNA transcripts in the form of produced cDNA corresponds with the size of predicted exons in both SOD and CAT genes of *C. themophilum* var. *dissitum* (details in Table S1). This was further confirmed with Sanger sequencing of all here presented cDNAs. Obtained complete DNA sequences of spliced SOD and CAT genes were submitted to GenBank (accession mumbers MW029961-MW029963). Moreover, in SOD samples after RT-PCR we see a slight induction of specific mRNA production (Figure 1; slots 2–3, 5–6, 11–12) upon addition of 2 mM hydrogen peroxide in the growth medium in comparison with control samples (where no H_2_O_2_ was added).

### 3.2. Superoxide Dismutases (SOD, EC 1.15.1.1) 

Superoxide dismutases are rather small but very compact metalloenzymes that are found in all kingdoms of life in a variety of forms with ancient origins. These proteins catalyze the dismutation of superoxide anion free radical (O_2_^−^) into molecular oxygen and hydrogen peroxide (H_2_O_2_, Equation (1)) and thus rapidly decrease the O_2_^−^ level, which damages aerobically metabolizing cells at excessive concentrations [23]. The emerging product, hydrogen peroxide, is the direct substrate of catalases that decompose it to harmless final products. The typical superoxide dismutase reaction is accompanied by cyclic oxidation and reduction of metal ions present in the active site of various SODs. According to the metal located at the active site of the enzyme, SODs could be divided into three types: copper/zinc-containing SOD (CuZnSOD), iron-containing SOD (FeSOD), and manganese-containing SOD (MnSOD). Each type exhibits different sensitivity to KCN, NaN_3_, and H_2_O_2_. There are also some novel SODs discovered in the past decades, such as cambialistic SOD and nickel SOD (NiSOD), the former could function well either with iron or manganese at its active site [24]. It is noteworthy to mention that a new subclass of copper containing SODs has recently emerged. Copper-only enzymes serve as extracellular SODs in specific bacteria (i.e., Mycobacteria), throughout the fungal kingdom, and also in oomycetes [25].

A rather high level of expression of CuZnSOD and MnSOD genes occurred under oxidative stress in the stationary growth phase in the model yeast *S. cerevisiae* [26]. Most native SODs are presented as highly stable, within a wide temperature range, and they are also resistant to inactivation within a wide range of pH. Thermostable SODs from various hyperthermophiles have already been reported for diverse aspects such as modified superoxide dismutase from the archaeon *Sulfolobus solfataricus* [27], thermostable SOD from *Bacillus licheniformis* SPB-13 originating in Himalayan region [28], and manganese-dependent superoxide dismutase in the Gram-negative bacterium *Thermus thermophilus* increasing tolerance to toxic metal ion [29]. There are apparently no known hyperthermophiles among eukaryotes, but unique MnSOD was already found in the thermophilic fungus *Chaetomium thermophilum* [30]. Moreover, a novel CuZnSOD originating from the same fungus but heterologously expressed in *Pichia pastoris* increased the antioxidant activity of the mesophilic yeast host [31]. Clearly, the thermostability is one of the most important properties that have been considered for potential biotech applications since thermal denaturation is a common cause of enzyme inactivation in industrial use [24]. We have performed a detailed genomic analysis in our newly discovered fungal genome and found two distinct forms of superoxide dismutase genes present regularly in genomes of thermophilic fungi. To demonstrate their peculiarities, we have reconstructed phylogenetic relationships for two independent gene families, namely CuZnSOD and FeMnSOD, respectively. The third (minor) family, namely NiSOD, was not found in the genome of any thermophilic fungus yet and it is spread only among bacteria.

#### 3.2.1. Copper-Zinc Superoxide Dismutases

**Figure 2:**
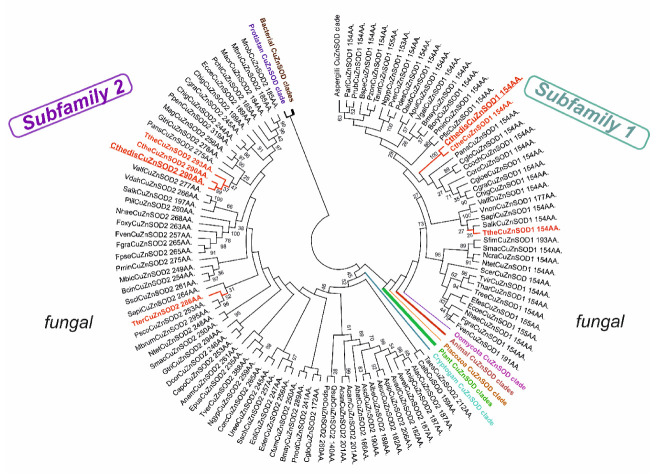
Global phylogenetic tree of the CuZnSOD family. This reconstruction was obtained with the maximum likelihood method of MEGA-X package [15] by using Whelan and Goldman model of substitutions. It contained 330 full length sequences of CuZnSODs. Partial deletion with 14% sites coverage and 100 bootstrap replications were used in this reconstruction. Only bootstrap values above 25 are shown. There were in total 207 amino acid positions used in the final dataset. Sequences from thermophiles are labeled in red. Abbreviations of used sequences are explained in Table S2.

The evolutionary relationships for CuZnSOD family with main focus on fungal genes are presented in Figure 1 in a circular form. In this robust maximum likelihood tree up to 312 full-length protein sequences were used. It is clear that in most fungi two distinct paralogs of this widespread gene family exist that are well separated. They must have occurred via an ancestral gene duplication event long before the ancient segregation of fungal kingdom from other eukaryotic kingdoms occurred. The thermostable representatives are present in both separated clades. For the first clade (CthedisCuZnSOD1) they are closely related with mesophilic counterparts from the class *Sordariomycetes.* Some of them are encoded in typical soil fungi but some even originate in fungi that are considered as pathogenic (e.g., *Scedosporium apiospermum* or *Colletotrichum higginsianum*). All representatives of Clade 1 have shorter sequences (in average only around 155 amino acids). The second clade (Figure 2 right part) contains more fungal thermostable variants and the proteins are generally much longer if compared with Clade 1 (in average above 250 amino acids). CthedisCuZnSOD2 from this clade is also closely related with some fungal mesophilic variants from the class *Sordariomycetes* like *Podospora anserina* or *Chaetomium globosum* that are considered as non-pathogenic soil saprotrophs. Thermophilic CuZnSOD2 are more distantly related with several ascomycetous SODs originating in phytopathogens (like SOD2 from *Sclerotinia sclerotiorum*).

**Figure 3:**
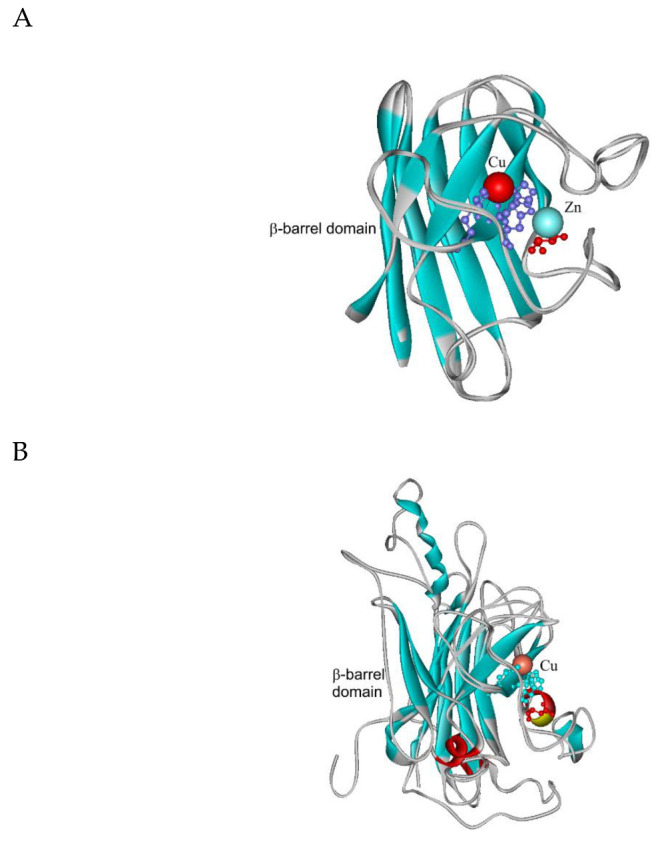
Structural models of two paralogous CuZnSODs from *Chaetomium thermophilum* var. *dissitum* modelled with Phyre-2 server [16]. (**A**) CthedisCuZnSOD1 modelled on the structure of 1f1g coding for ScCuZnSOD1 (from *Saccharomyces cerevisiae*). (**B**) CthedisCuZnSOD2 modelled on the structure of 4n3u coding for CalbCuSOD2 (from *Candida albicans*).

In the multiple sequence alignment (Figure S2) we can observe a high level of conservation in the active center—mainly for essential histidines involved in copper binding but not for all of histidines that are supposed to bind zinc. This peculiarity is typical mainly for Clade 2 CuZnSOD representatives that apparently could effectively bind only Cu ions to fulfil the superoxide dismutation reaction [32]. It is also apparent that Clade 2 members exhibit several insertions along their sequences between regions responsible for metal binding that explain their bigger size. Structural homology models for thermophilic CuZnSODs are presented in Figure 3. Homology model of newly discovered CthedisCuZnSOD1 in a structural overlay with closely related known experimental structure of yeast CuZnSOD1 showing the position of Cu and Zn ions is presented in Figure 3A. In principle this short protein only consists of a rather compact and highly conserved β-barrel domain. In contrary, the structural overlay of CthedisCuZnSOD2 with its closest homolog with known experimental 3D structure (CuSOD5 from *Candida albicans*) exclusively shows the presence of Cu ions in the active center (Figure 3B). Moreover, besides the central β-barrel domain it also contains several quite long loops and also few short α-helices. Thus, the evolutionary segregation of CuZnSODs in two paralog clades is supported also with significant differences in the homology models of their respective 3D structures. Most known Clade 1 representatives are physiologically homodimeric or tetrameric enzymes. In contrast, some Clade 2 representatives were shown to be only monomeric. Furthermore, with respect to their subcellular locations Clade 1 is supposed to contain intracellular, largely cytosolic proteins. On the other hand, Clade 2 is represented mainly by extracellular enzymes [30]. The sequences from newly sequenced thermophilic genome fully confirm this rule: CthedisCuZnSOD1 is intracellular and CthedisCuZnSOD2 is extracellular with a high probability as predicted with SignalP-5.0 server.

#### 3.2.2. Iron-Manganese Superoxide Dismutases

**Figure 4:**
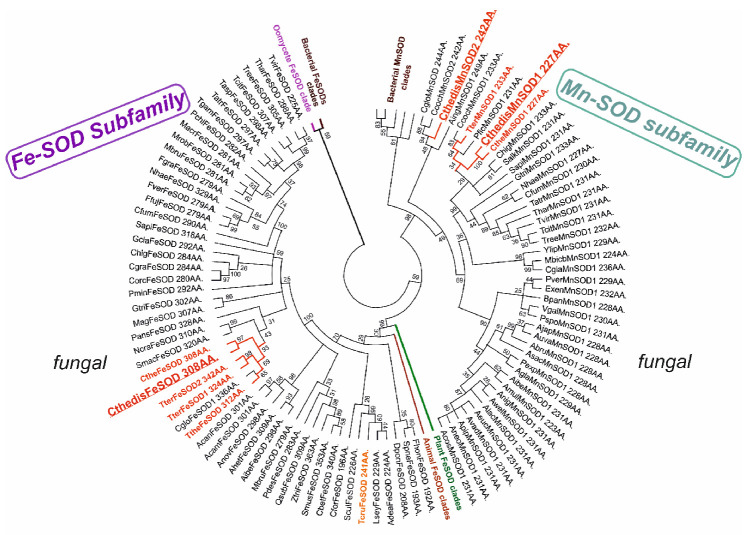
Global phylogenetic tree of 524 members of the Fe-MnSOD family obtained with the Maximum Likelihood method of MEGA-X package [15] by using Le Gascuel model of substitutions. Partial deletion with 38% sites coverage and 100 bootstrap replications were used in this reconstruction. Only bootstrap values above 25 are shown. There were in total 223 amino acid positions used in the final dataset. Sequences from thermophiles are labeled in red. Abbreviations of used sequences are explained in Table S3.

**Figure 5:**
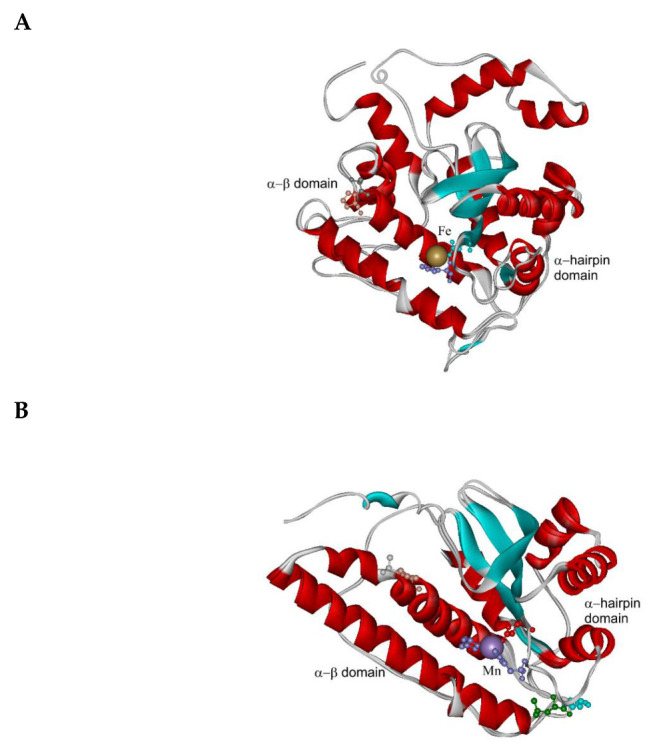
Structural models for members of the Fe-MnSOD family from *Chaetomium thermophilum* var. *dissitum* modeled with Phyre-2 server [16]. (**A**) FeSOD modeled on the structure of 4h3e from *Trypanosoma cruzi*, presented in Figure 4 as TcruFeSOD and (**B**) MnSOD1 modeled on the structure of 4br6 from *Chaetomium thermophilum* var. *thermophilum* presented in Figure 4 as CtheMnSOD.

The evolutionary relationships for FeMnSOD family with main focus on fungal genes are presented in Figure 4 in a circular form. From this global evolutionary tree obtained with the maximum likelihood method and comprising up to 524 full-length sequences a clear separation between FeSOD and MnSOD clades is unequivocal. MnSOD are a bit shorter (in average around 230 amino acids) than FeSOD (in average around 290 amino acids) but the differences are not as prominent as observed in the CuZnSOD family. Also, in the case of FeSOD the thermophilic variants are closely related with mesophilic soil counterparts from the class *Sordariomycetes* like *Chaetomium globosum, Neurospora crassa* or *Podospora anserina.* They are more distantly related with ascomycetous phytopathogenic FeSODs like *Magnaporthe oryzae* or *Gauemannomyces tritici* (causing take-all root disease in wheat and barley). Thermophilic manganese superoxide dismutases are likewise closely related with mesophilic soil counterparts from the class *Sordariomycetes* represented by *Chaetomium cochliodes* or by another interesting extremophile *Sodiomyces alkalinus* [33]. Phytopathogenic ascomycetous representatives e.g., MnSOD from *Gauemannomyces tritici* are more distantly related to them. The differences in the primary structure of FeSOD vs. MnSOD can be clearly seen from the multiple sequence alignment presented in Figure S3. In general, MnSODs reveal higher level of overall sequence conservation and FeSOD exhibit more variations within their clade and short insertions in comparison with MnSOD. Structural differences among these two distinct clades of such a large gene family are surprisingly not as pronounced as in CuZnSOD family. The highly conserved fold typical for the whole FeMnSOD family is presented in Figure 5. It appears that FeSOD structural model (Figure 5A) is more robust where multiple α-helices and several loops can be found around the conserved active centre located in the α-β domain. In contrast, MnSOD structural model (Figure 5B) appears to be more compact with conserved and connected α-hairpin and α-β domain. Moreover, it is known that under physiological conditions it forms higher assembly structures—homodimers or mainly homotetramers [34]. In contrast, FeSODs tend to form homodimers (e.g., FeSOD structure with PDB code 4H3E and [23]) but this needs an experimental proof for fungal representatives. Most of eukaryotic MnSODs are located in mitochondria whereas FeSOD isozymes can be distributed between mitochondria and cytosol. In the case of sequences from the newly sequenced thermophilic genome: both CthedisFeSOD and CthedisMnSOD are located in mitochondria with a high probability as predicted with TargetP-2.0 server.

### 3.3. Catalases (CAT, EC 1.11.1.6) 

Catalases are registered in enzyme databases as hydrogen-peroxide:hydrogen peroxide oxidoreductases. They are frequently occurring antioxidant enzymes present in both prokaryotic and eukaryotic cells that can both reduce and oxidize H_2_O_2_. They can be divided into heme and nonheme catalases. Nonheme (manganese) catalases were not detected among eukaryotes yet and are not the focus of this research. All heme catalase superfamily members function in the rapid decomposition of H_2_O_2_ to harmless products (cf. Equation (3)). Hydrogen peroxide is a frequent by-product either from the SOD-catalyzed reaction or also a by-product of various oxidases or it can occur in xenobiotic mixtures from extracellular environmental stress. The enzyme uses heme (i.e., ferriprotoporphyrin IX) as a cofactor and catalyzes the heterolytic cleavage (comprising a reduction and concomitant oxidation cycle) of hydrogen peroxide to water and molecular oxygen, thus completing the cellular detoxification process initiated by various SODs. Catalases react in this way by finally limiting the effective peroxide concentration to physiologically acceptable levels [35]. Most of heme catalases in fungi belong to the largest superfamily of typical catalases that were called monofunctional catalases before [36]. Although their predominant activity is the above mentioned heterolytic cleavage of hydrogen peroxide, they can also possess some minor peroxidase activity according to Equation (4):

H_2_O_2_ + 2AH_2_ → 2 H_2_O + 2 HA* (A—donor of electrons) (4)

It shall be noted that this reaction scheme is typical for all kinds of peroxidases and “A” in this equation can be numerous 1- or 2-electron donors of various types e.g., methanol, ethanol, formic acid, or phenols and their substituted derivatives as well as aromatic amines [37]. Bifunctional enzymes named catalase-peroxidases (abbreviated as KatGs) also exist (E.C. 1.11.1.21) [36]. They are able to react in both catalatic and peroxidatic modes. If reacting as peroxidases, they can be involved in polymerization reactions [38]. These unique enzymes are also present among fungi (mainly among Ascomycetes) but they are physiologically not as dominant as typical catalases. Moreover, their real physiological substrate is still the matter of debate. 

Although there are several differences in the primary structure among numerous typical heme catalases, the unique three-dimensional structural fold appears to remain well conserved [39]. Catalase counts to the most efficient natural enzymes known; it can break down millions of hydrogen peroxide molecules in just one second under physiological conditions. This antioxidant enzyme is among most eukaryotes located primarily in specific organelles known as peroxisomes [40]. It was claimed that heme catalase is absent in mitochondria of at least mammalian cells where its role can be replaced with specialized glutathione peroxidase. Curiously, mitochondrial-targeted catalase was described recently exhibiting positive effects on life span and health span extensions in laboratory animals [3]. 

**Figure 6:**
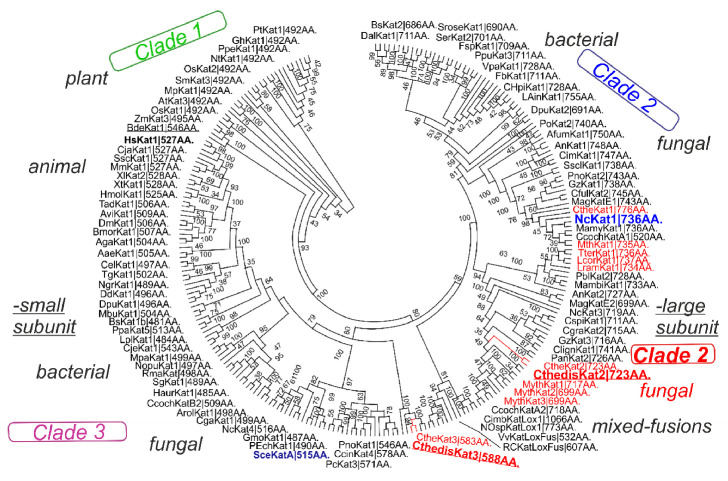
Global phylogenetic tree of 250 heme catalases obtained with the maximum likelihood method of MEGA-X package [15] by using Le Gascuel model of substitutions. A discrete Gamma distribution to model evolutionary rate difference, partial deletion with 33% site coverage, and 100 bootstrap replications were used in this reconstruction. Only bootstrap values above 30 are shown. There were in total 694 positions used in the final dataset. Sequences from thermophiles are labeled in red. Abbreviations of used sequences are explained in Table S4.

**Figure 7:**
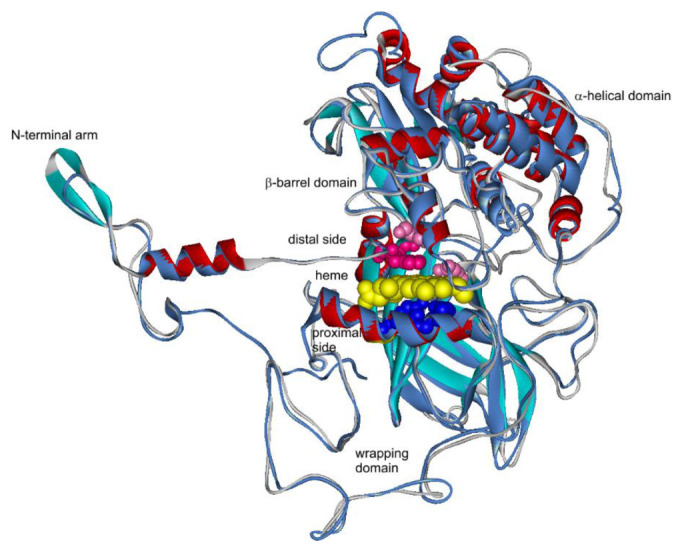
Structural model of a thermophilic heme catalase 2 secreted from *Chaetomium thermophilum* var. *dissitum* modeled with Phyre-2 server [16]. This structure was superimposed with MultiProt server [17] to the experimentally obtained structure with PDB code 1SY7 that represents NcKat1 of mesophilic *Neurospora crassa* [6].

Microbial catalases are still preferred in most biotechnologies due to their economic feasibility, high production yield, ease of product modification and optimization, regular supply due to absence of seasonal fluctuations, and rapid growth of microbes on an inexpensive media. Most preferred are catalases produced from alcali-thermophilic microorganisms because of their ability to withstand high temperature and pH conditions. Numerous thermostable catalases have already been found in extremely thermophilic bacteria e.g., *Thermus thermophilus* HB8 [41], *Geobacillus* sp. CHB1 [42], *Geobacillus thermopakistaniensis* [43], and *Deinococcus radiodurans* [44] but in fungi they are still rather rare. In this contribution, we describe heme catalases from the thermophilic fungus *Chaetomium thermophilum* var. *dissitum* [45]. We have reconstructed the updated molecular phylogeny of typical catalases by adding newly discovered sequences with maximum likelihood method in Figure 6. It is a robust circular tree with 250 full-length protein sequences mainly focused on fungal representatives. The previous division of all catalases in three main evolutionary clades [46] can be clearly seen in this updated and extended presentation. Whereas Clade 1 is dominantly formed by plant catalases, in both Clades 2 and 3, numerous bacterial, fungal, and (for Clade 3) also animal catalases are involved. Furthermore, in basic 3-clades division, a clear separation between small-subunit and large-subunit heme catalases is also obvious from the tree presented in Figure 6. Clades 1 and 3 together contain only small-subunit catalases with an average size of 510 amino acids. In contrast, the large-subunit catalases typical for Clade 2 have an average size of over 720 amino acids and thus are much more complex. Additionally, only this clade contains enzymes that contain heme *d* besides (much more frequently occurring) heme *b* as the prosthetic group in their active centers [6]. As obvious from Figure 6, Clade 2 is very abundant on various fungal representatives and contains both mesophilic and thermophilic enzymes (labeled red in Figure 7) that are closely related. Moreover, the small-subunit catalases are predominantly intracellular, mostly containing peroxisomal targeting signals, and the large-subunit catalases are predominantly extracellular, irrespective of their origin as confirmed with the above already mentioned SignalP-5.0 server. This different cellular localization feature has apparently very old evolutionary descent.

Multiple sequence alignment of selected heme catalase sequences is presented in Figure S4 and shows the essential residues responsible for the typical mechanism of peroxide diffusion and heterolytic bond cleavage. On the distal side of prosthetic heme group (Figure S4A), there are essential His, Ser, and Asn that are invariantly conserved together with parts of the substrate channel formed by bulky Val and two neighboring Phe. On the proximal heme side (Figure S4B), there are invariant Tyr and Arg that are also highly conserved in all functional members of this superfamily and are responsible for the correct orientation of the prosthetic heme group.

Homology model of CthedisKat2—representing the extracellular large-subunit catalase—is presented in Figure 7. It clearly shows the highly conserved catalase fold of a monomer with heme. Under physiological conditions, these monomers fold in a compact homotetrameric assembly by hooking the N-terminal arm into the wrapping domain [6].

### 3.4. Outlook and Future Perspectives

Superoxide dismutases and catalases constitute a combination of physiologically complementary enzymatic antioxidants appropriately called “first line defense antioxidants” [33]. They both act very effectively to suppress or prevent the formation of free radicals or reactive catabolic species in the cells from which they originate or in their close environment. From our analyses presented in this contribution, it is apparent that superoxide dismutases, mainly representatives of FeMnSOD family, reveal much more diversity in comparison with the superfamily of heme catalases. The evolutionary reconstruction indicates certain common features in the evolution of all here presented gene families. Mainly, a close phylogenetic relationship between SOD genes as well as catalase genes from thermophilic Ascomycota and soil mesophilic Ascomycota was observed (Figure 1, Figure 4 and Figure 7, respectively). Additionally, a more distant relationship between these genes from thermophilic Ascomycota and phytopathogenic fungi was also detected. One interesting aspect is that, in parallel with the general reduction of genome size between mesophilic and thermophilic fungal genomes (Figure S1 and [20,21]), a reduction in the number of genes for catalase can be observed. This can be prominently followed within the family of *Chaetomiaceae*. Whereas the mesophilic *Chaetomium cochliodes* contains up to four genes for typical catalases (one pair for small-subunit and one pair for large-subunit catalase), the count of these genes is reduced to three in *Chaetomium thermophilum* var. *thermophilum*. But in *Chaetomium thermophilum* var. *dissitum* (although very closely related), one gene for a large subunit catalase revealing some deletions (Figure S4) was probably lost very recently, resulting in only two functional catalase genes (one small- and one large-subunit variant). In contrast, the total count of both CuZnSOD and FeMnSOD remains the same in both mesophilic and thermophilic Ascomycetes.

The potential application of both these physiologically closely connected enzymatic antioxidants is promising in both medicine and biotechnology. As already well documented for both recombinant yeasts [31,34] and mammalian cell lines [3], enhanced heterologous expression of antioxidant enzymes leads to increased health and life span via acquired higher stress resistance. Thus, it is logical to consider a future design of engineered variants based on rational approach from known 3D structures of both types of antioxidant enzymes. To adapt the existing evolutionary conserved structural patterns and folds for specific purposes can be a quite challenging task. The designer fusion of SOD with human hemoglobin [47] can serve as a good starting model. It is known that large subunit catalases contain an additional flavodoxin-like domain [6] that has no catalytic function. A similar idea to [47] would suggest replacing this domain in a thermostable catalase with a short thermostable CuZnSOD1 or MnSOD domain of comparable length. The overall stability of this construct needs to be verified in the future. 

## 4. Conclusions

We have reconstructed detailed molecular phylogeny of three important gene families coding for main antioxidant enzymes with focus on thermophilic eukaryotes. We can conclude that there are similar and conserved evolutionary patterns mainly between the phylogeny of fungal CuZnSOD family and fungal heme catalase superfamily. A designer protein fusion between large subunit heme catalase and a superoxide dismutase based on analyzed sequences here is suggested. It is based on replacement of a noncatalytic domain in large thermostable catalase with an appropriate thermostable CuZnSOD1 or MnSOD domain of comparable length. A close interplay of thermostable SOD and CAT is attractive for consideration in future medical and biotech applications.

## Figures and Tables

**Table 1:** List of important genes (either barcode-specific or antioxidant-coding) detected in the genome of *Chaetomium thermophilum* var. *dissitum* from the genome project JAAFKN000000000.

Gene	NODE No., NG1-Length	NG1-Gene Position:START	NG1-Gene Position:END	NG1-Segment Length [bp]	NODE No., NG2-Length	NG2-Gene Position:START	NG2-Gene Position:END	NG2-Segment Length [bp]
**18S-5.8S-28S**	NODE 965, 8730bp	2845	8481	5637	NODE 1882, 4524bp NODE 3062, 3145bp	1800 2671	4524 5637	2725 2967
**Tubulin**	NODE 244, 16828bp	10315	12185	1871	NODE 481, 8751bp	2626	4496	1871
**Cthedis_rpb2**	NODE 1953, 4913bp	1825	4913	3089 *	NODE 476, 8765bp	5597	8734	3138
**CthediskatG1**	NODE 890, 9086bp	1133	3743	2611	NODE 2179, 4103bp	1059	3669	2611
**Cthediskat2**	NODE 1031, 8272bp	4055	7080	3026	NODE 785, 7192bp	1345	4370	3026
**Cthediskat3**	NODE 1987, 4805bp NODE 1207, 7396bp	3369 1	4805 949	1437 949	NODE 933, 6623bp	1062	3392	2331
**CthedishyBpox1**	NODE, 1039, 8260bp	4218	7654	3437	NODE 1837, 4610bp	430	3866	3437
**CthedisCuZnSOD1**	NODE 1294, 7102bp	915	1809	895	NODE 538, 8395bp	2187	3081	895
**CthedisCuZnSOD2**	NODE 986, 8616bp	1	952	952 *	NODE 3860, 2496bp NODE 1131, 6027bp	49 5957	881 6027	833 * 71 *
**CthedisFeSOD**	NODE 3217, 2500bp	748	1742	995	NODE 4012, 2388bp	659	1653	995
**CthedisMnSOD**	NODE 15, 36954bp	10874	11747	874	NODE 769, 7230bp	4132	5005	874

* partial = gene occurs only partially in the node.

## Supplementary Materials

The following are available online at https://www.mdpi.com/2076-3921/9/11/1047/s1, Figure S1. Main features of the newly sequenced genome of *Chaetomium thermophilum* var. *dissitum*. Figure S2. Multiple sequence alignment of CuZnSOD. Figure S3. Multiple sequence alignment of FeMnSOD. Figure S4. Multiple sequence alignment of heme catalases. Table S1. Sequences of DNA primers used for PCR analyses. Table S2. Sequences of CuZnSOD used for phylogenetic reconstruction with their accession numbers and taxonomic origin. Table S3. Sequences of FeMnSOD used for phylogenetic reconstruction with their accession numbers and taxonomic origin. Table S4. Sequences of heme catalases used for phylogenetic reconstruction with their accession numbers and taxonomic origin.
