# Supplementary material for: Parallel Molecular Evolution of Catalases and Superoxide Dismutases—Focus on Thermophilic Fungal Genomes
Source: Antioxidants (Basel). 2020 Oct 27;9(11):1047. doi: 10.3390/antiox9111047 (PMC7712995; doi:10.3390/antiox9111047)
Supplement: Supplementary file 1 [file antioxidants-09-01047-s001.zip › Supplementary Figure 1 Report about genome of Chaetomium thermophilum dissitum.pdf]

Report

	contigs
# contigs (>= 0 bp)	15210
# contigs (>= 1000 bp)	4552
# contigs (>= 5000 bp)	1932
# contigs (>= 10000 bp)	765
# contigs (>= 25000 bp)	67
# contigs (>= 50000 bp)	2
Total length (>= 0 bp)	29434923
Total length (>= 1000 bp)	27455616
Total length (>= 5000 bp)	20583149
Total length (>= 10000 bp)	12345051
Total length (>= 25000 bp)	2142392
Total length (>= 50000 bp)	106138
# contigs	5452
Largest contig	54257
Total length	28100611
GC (%)	53.47
N50	8792
N75	4715
L50	948
L75	2034
# N's per 100 kbp	0.00

All statistics are based on contigs of size >= 500 bp, unless otherwise noted (e.g., "# contigs (>= 0 bp)" and "Total length (>= 0 bp)" include all contigs).

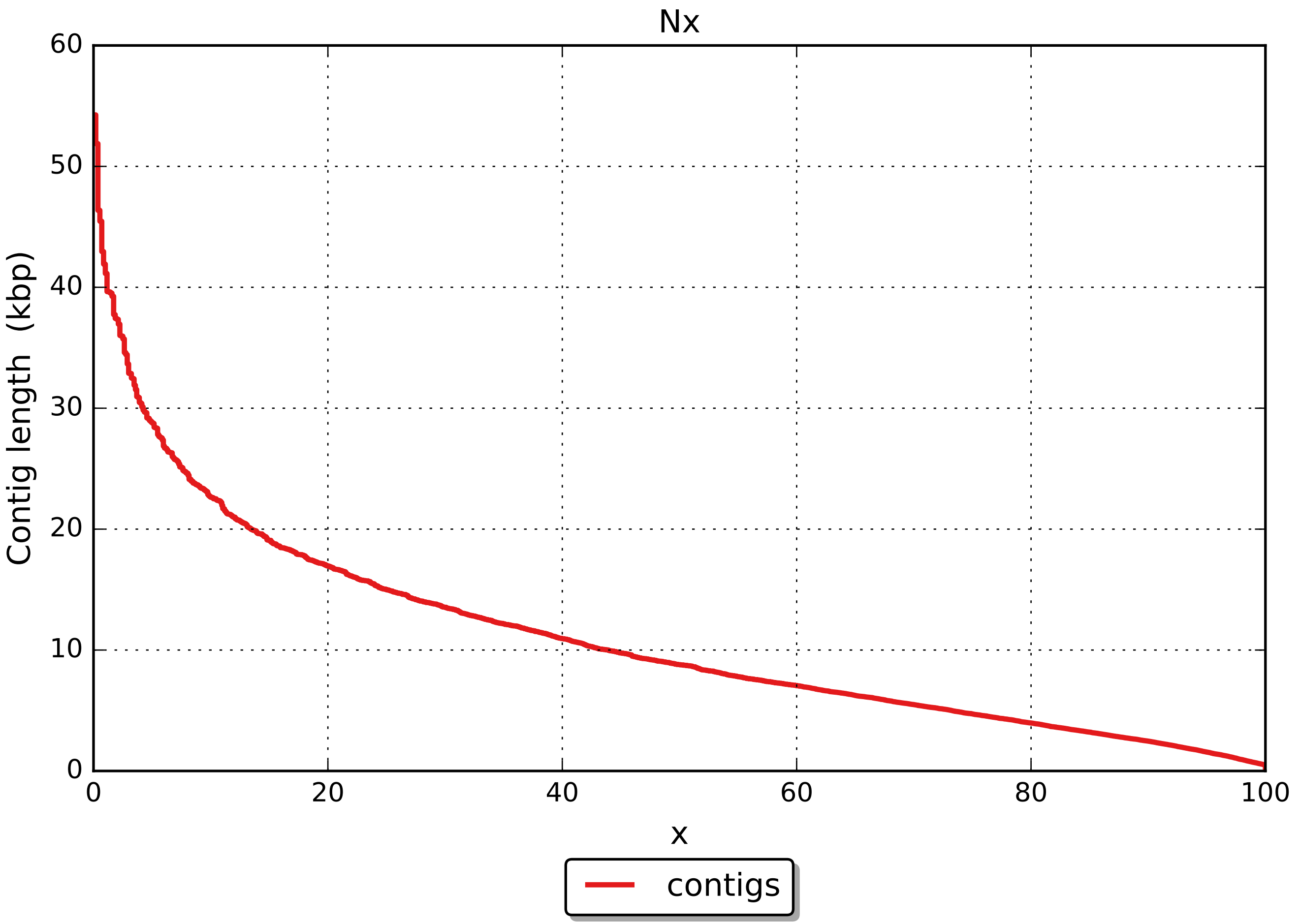

Nx
Contig length (kbp)
x
0
10
20
30
40
50
60
0
20
40
60
80
100
contigs

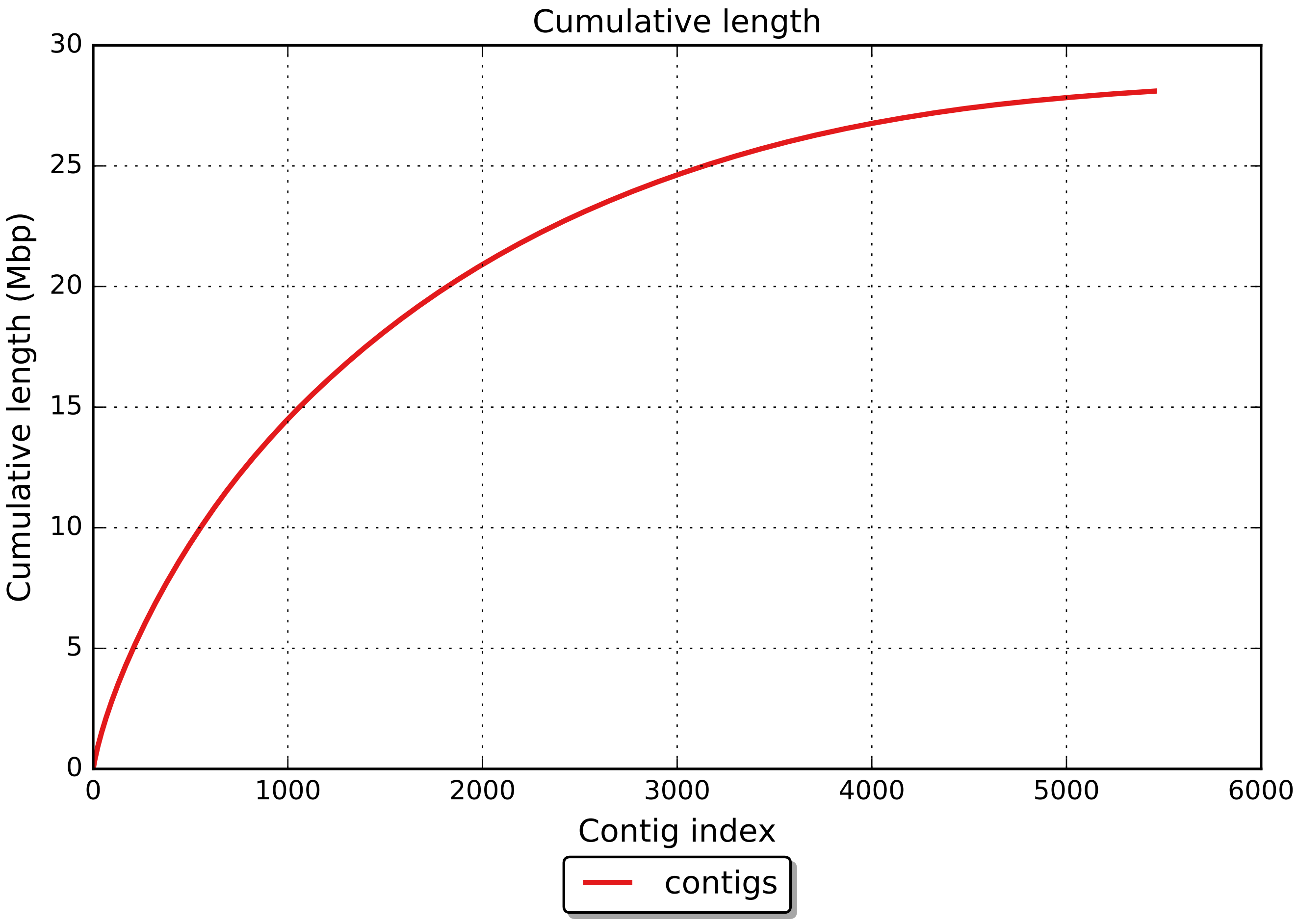

Cumulative length
Cumulative length (Mbp)
0
5
10
15
20
25
30
0
1000
2000
3000
4000
5000
6000
Contig index
contigs

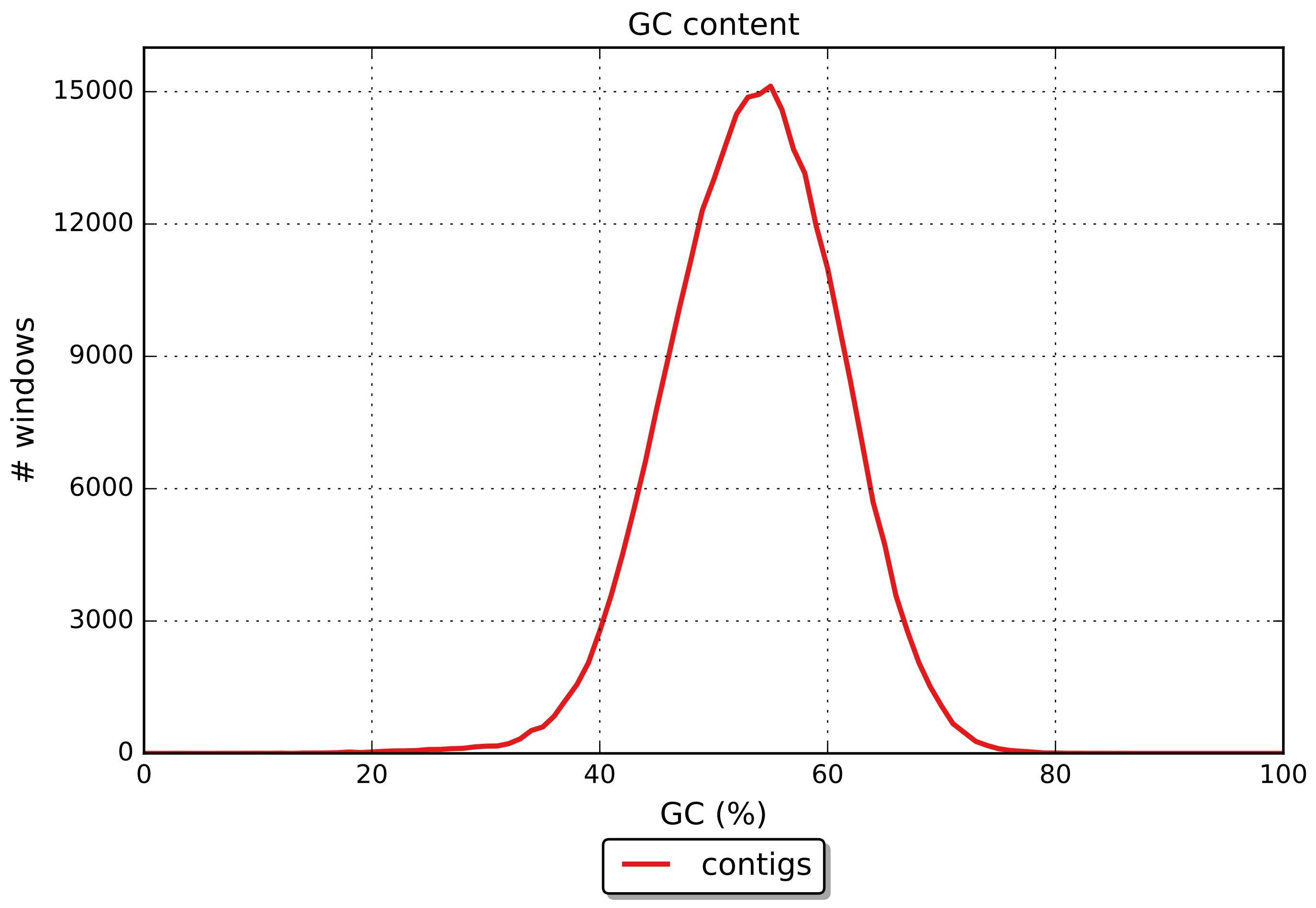
GC content
15000
12000
9000
6000
3000
0
windows
0
20
40
60
80
100
GC (%)
contigs

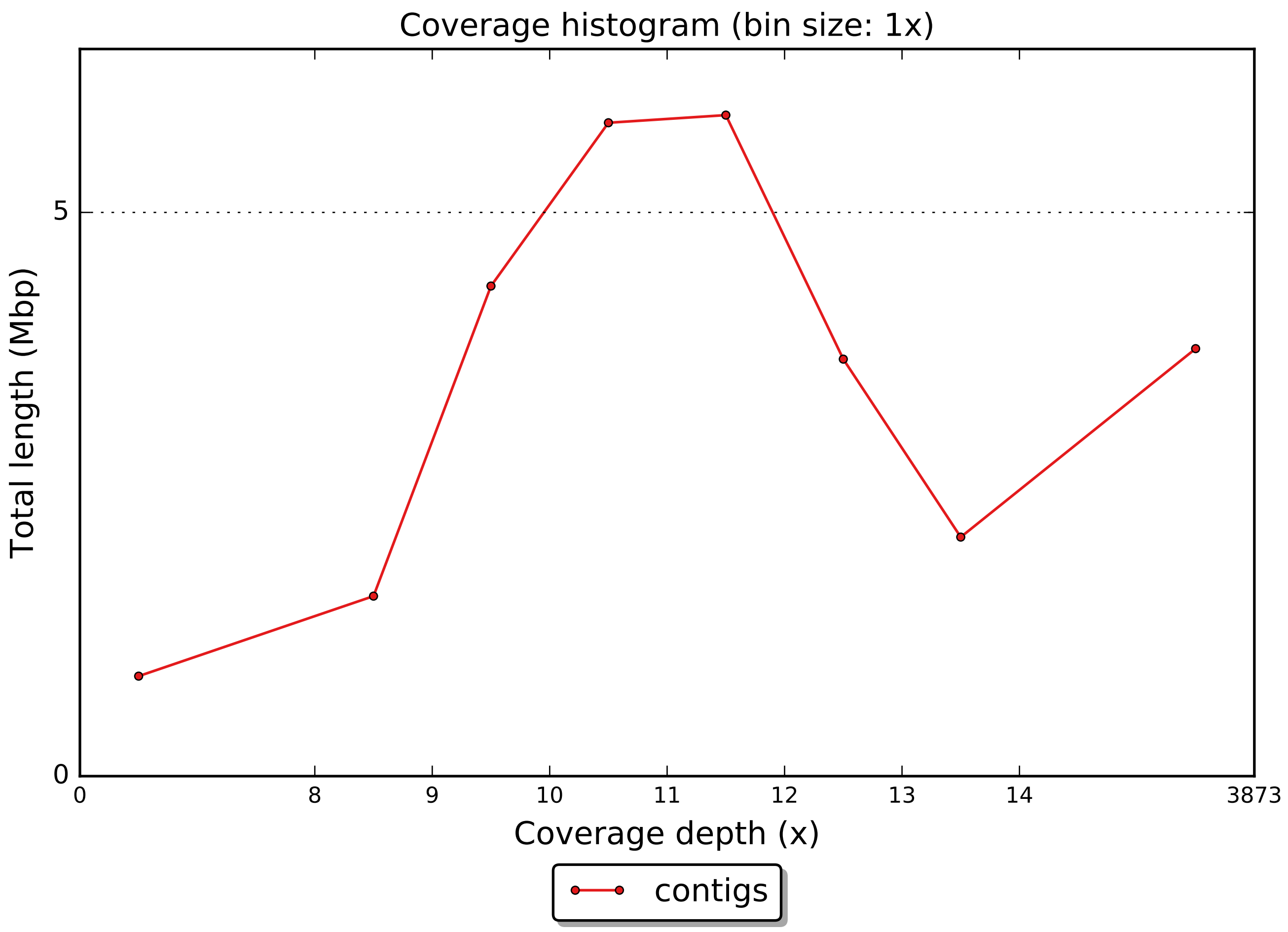

Coverage histogram (bin size: 1x)
Total length (Mbp)
5
0
0
8
9
10
11
12
13
14
3873
Coverage depth (x)
contigs

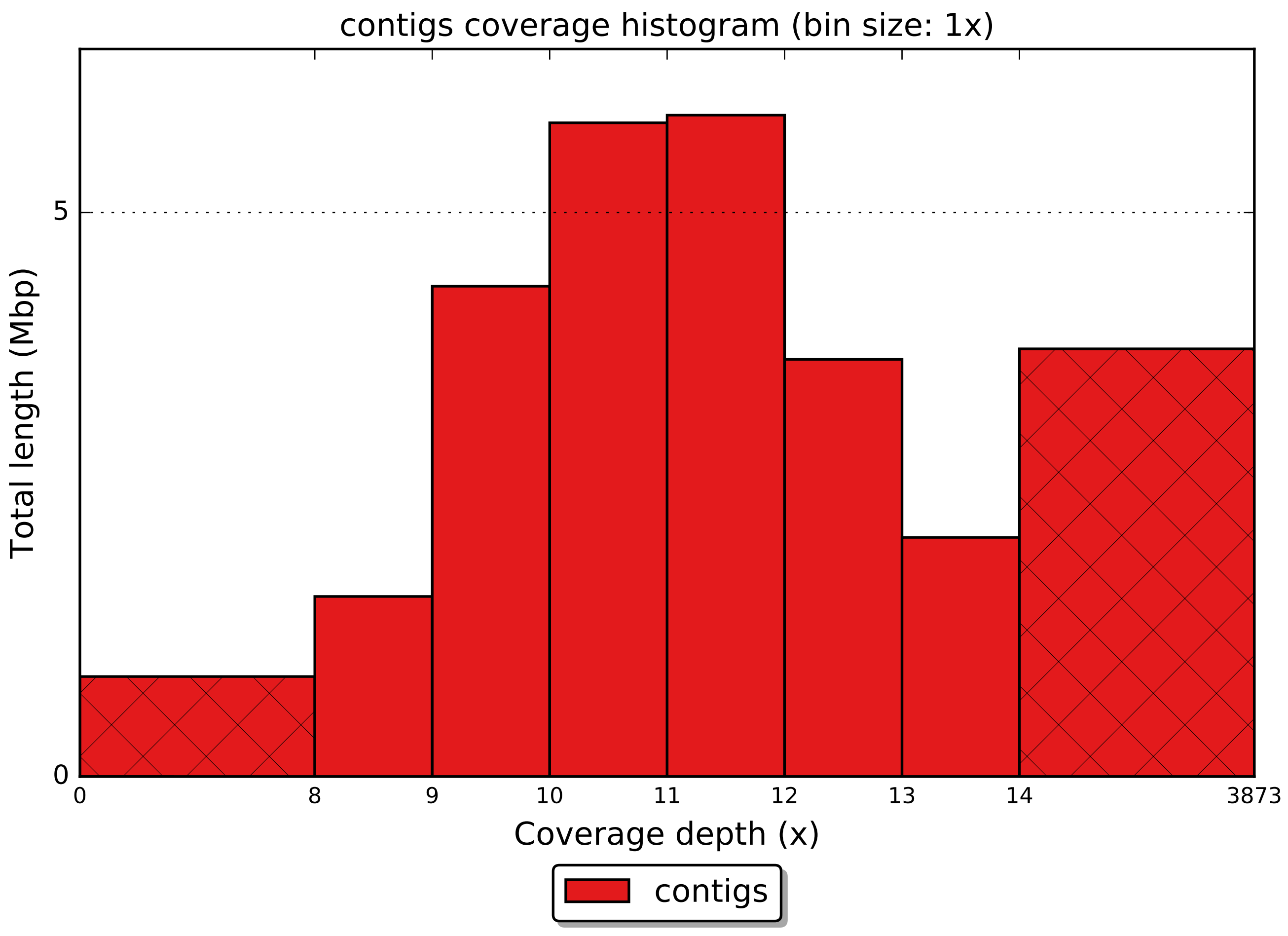
contigs coverage histogram (bin size: 1x)
Total length (Mbp)
5
0
0
8
9
10
11
12
13
14
3873
Coverage depth (x)
contigs
