# Supplementary material for: Parallel Molecular Evolution of Catalases and Superoxide Dismutases—Focus on Thermophilic Fungal Genomes
Source: Antioxidants (Basel). 2020 Oct 27;9(11):1047. doi: 10.3390/antiox9111047 (PMC7712995; doi:10.3390/antiox9111047)
Supplement: Supplementary file 1 [file antioxidants-09-01047-s001.zip › Supplementary Figure 2 (CuZnSOD alignment).pdf]

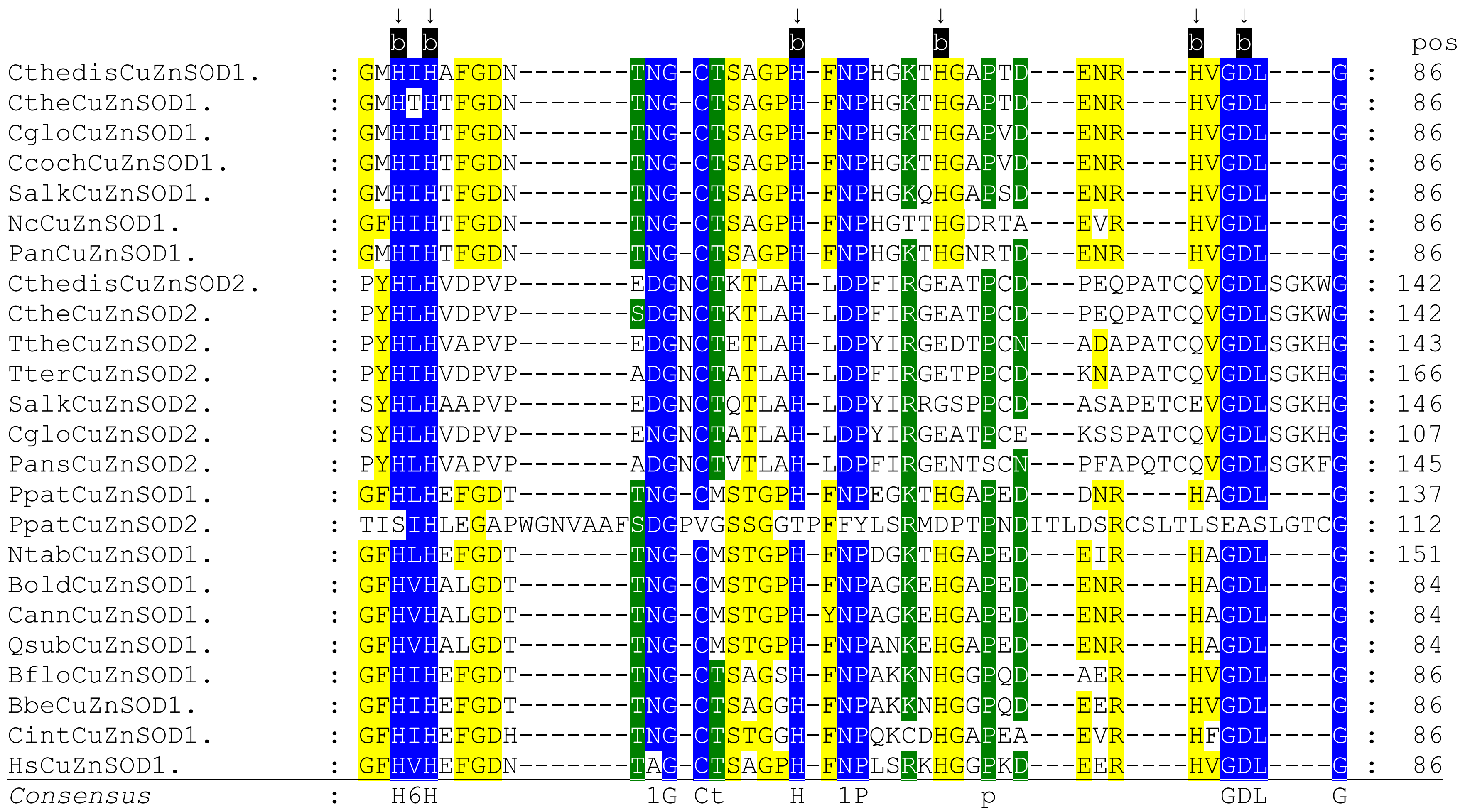

Suppl. Figure 2. Multiple sequence alignment showing essential regions of CuZnSOD obtained with Muscle implemented in MEGA X package [14]. Highly conserved regions responsible for metal binding are shown above the alignment. Color scheme: blue - highly conserved positions (above 95% of all aligned sequences), green - moderately conserved (between 75-95%), yellow - weakly conserved positions (between 50-75%). Numbers on the right represent corresponding amino acid position of each sequence.

```
                                                    ↓
                                                    b                   pos
CthedisCuZnSOD1.   : NIETDANGNSKGTMTDHLVK-LIGPESVIG-RTVVVHAGT-------------D--------- : 125
CtheCuZnSOD1.      : NIETDANGNSKGTMTDHLVK-LIGPESVIG-RTVVVHAGT-------------D--------- : 125
CgloCuZnSOD1.      : NIETDAQGNSKGTVTDKHIK-IIGPESVIG-RTVVVHAGT-------------D--------- : 125
CcochCuZnSOD1.     : NIETDAQGNSKGSVTDKHIK-IIGPESVIG-RTVVVHAGT-------------D--------- : 125
SalkCuZnSOD1.      : NIETDAQGNSKGEVTDSLIK-LIGPESVIG-RTVVVHAGT-------------D--------- : 125
NcCuZnSOD1.        : NIETDAQGNAKGTVTDNLVK-LIGPESVIG-RTVVVHAGT-------------D--------- : 125
PanCuZnSOD1.       : NIETDAQGNSKGTVTDNLIK-LIGPESVIG-RTVVVHAGT-------------D--------- : 125
CthedisCuZnSOD2.   : DIIPDENGTFEQTYTDLYASTKEGIGAFFGNRSVVVHFPN-------------KTRITCASF : 191
CtheCuZnSOD2.      : DIIPDENGTFEQTYTDLYASTKEGIGAFFGNRSIVVHFPN-------------K-TRITCAN : 190
TtheCuZnSOD2.      : KIPANKT-TFETSYVDLYASTVEGIGAFFGNRSIVFHFPN-------------K-TRITCAN : 190
TterCuZnSOD2.      : AIPTDQD-TWETSYVDLYASTLEGIGAFFGNRSIVVHYPN-------------K-TRITCAS : 213
SalkCuZnSOD2.      : LIDP-AGGAFEAVYVDEFASLIEGQGAFFGNRSFVLHLAS-------------D--------- : 186
CgloCuZnSOD2.      : AITPDAEGAFEATYVDLYASTLEGIGAFFGNRSIVFHHPN-------------K-TRISCAS : 155
PansCuZnSOD2.      : EIRPEEDGTWETTYTDLYSSTLEGLGSFFGNRSIVFHYPN-------------K-TRISCAN : 193
PpatCuZnSOD1.      : NVIAGNDGVVEVTLEDSQIP-LSGPHSVVG-RAFVIHEAE-------------D--------- : 176
PpatCuZnSOD2.      : SVDPENPTCARLTLSGKMMEITDQQELDFAALALFSKHPEMPDWPKWHKWIFYKLDILNIYL : 174
NtabCuZnSOD1.      : NIVANANGVAEATFVDNQIP-LTGPNSVVG-RALVVHELE-------------D--------- : 190
BoldCuZnSOD1.      : NVTAGADGVANVNVVDSQIP-LTGPQSIIG-RAVVVHADP-------------D--------- : 123
CannCuZnSOD1.      : NITVGEDGTASFTITDEQIP-LTGPQSIIG-RAVVVHADP-------------D--------- : 123
QsubCuZnSOD1.      : NVNVGADGTVNFTITDKQIP-LSGPNSIIG-RAVVVHADP-------------D--------- : 123
BfloCuZnSOD1.      : NVEVGDDGVATINITDSQLQ-LTGPNSIVG-RAVVVHAGE-------------D--------- : 125
BbeCuZnSOD1.       : NVEVGEDGEANIKITDSQLQ-LTGPNSIIG-RAVVVHAGE-------------D--------- : 125
CintCuZnSOD1.      : NVTADSSGVAEVNISDKYVT-LTGINSVIG-RAVVVHADV-------------D--------- : 125
HsCuZnSOD1.        : NVTADKDGVADVSIEDSVIS-LSGDHCIIG-RTLVVHEKA-------------D--------- : 125
Consensus          :   6     g       D       G     G R  V H
```

Suppl. Figure 2 continued.
