# Supplementary material for: Parallel Molecular Evolution of Catalases and Superoxide Dismutases—Focus on Thermophilic Fungal Genomes
Source: Antioxidants (Basel). 2020 Oct 27;9(11):1047. doi: 10.3390/antiox9111047 (PMC7712995; doi:10.3390/antiox9111047)
Supplement: Supplementary file 1 [file antioxidants-09-01047-s001.zip › Supplementary Figure 3 (FeMnSOD alignment).pdf]

```
                                        ↓↓           ↓
                                        bb           b                               pos
CthedisFeSOD1.    : DFEARQVKDILLMTAREPSQAPLFNYASMIHNNA-FFFKHLSP----EPVAMPE--PLKLHL : 139
CtheFeSOD1.       : DFEARQVKDILLMTAREPSQAPLFNYASMIHNNA-FFFKHLSP----EPVAMPE--HLKLHL : 139
TtheFeSOD.        : DYESRHVKDIMLSTARDPASAPLFNYASMIHNNH-FFFKHLTP----KPVEMPD--ALRVHL : 137
TterFeSOD1.       : EWESRHVKDIVLGTARDPNHAPTFNYASMIHNNH-FFFKHLSP----KPVKMPE--VLRGHL : 140
CgloFeSOD1.       : DYESHQIKDILLGTARDPGSAPIFNHASMAHNNH-FFFKHISP----KPVEVPE--PLRSRL : 137
CgloFeSOD2.       : -VAAVASDDV----AGQIALQQVIKFNGGGHINHSLFWKNLAPAGSNEANLDRA-SSLVAEI : 139
CcochFeSOD1.      : -VAAVASDDV----AGQIALQQVIKFNGGGHINHSLFWKNLAPAGSSEASLDRA-GGLVAEI : 137
PinfFeSOD.        : -KGAALKGKSILEVVQSATEAPVRN-NGGGHYNHSLFWTWMTS--PGSTNTAPH-GALKTRI : 103
DeleFeSOD.        : ELQGKSLDEIILAVKGDSSQAPVFN-NAGQHWNHILFWQNLSP----AGGAIPD--KLAAKI : 103
DponFeSOD1.       : ELEGKTLEEIITATAGDASKAGVFN-NAAQVWNHTFYWNCMAK----NGGGKAT-GTLAAKI : 103
PequFeSOD.        : SLYGSTLEELVKTTYNNGNPLPEFN-NAAEAWNHDFFWESMQP----DSGSMPW-GSLLHQI : 172
ZaetFeSOD.        : ELAGLSLEEIVIKSYNEGDLLPTFN-NAAQIWNHDFFWQSMKP----DGGGKPF-GVLMELI : 145
CthedisMnSOD1.    : -SEAEAKGDF----TKAASLAPLLNFHGGGHLNHTLFWENLAPA-SREGGGEPD-GALKKAI : 130
CtheMnSOD1.       : -AEAEAKGDF----TKAASLAPLLNFHGGGHLNHTLFWENLAPA-SREGGGEPD-GALKKAI : 130
TterMnSOD1.       : -AEAESKGEF----TKAASVAPLLNFHGGGHVNHSLFWENLAPA-NREGGGEPD-GALKKAI : 136
CcochMnSOD1.      : -GEAESKGDF----TKAASVAPLLNFHGGGHINHSLFWENLAPA-SREGGGEPD-GALKKAI : 136
PexpMnSOD1.       : -QEAQAKGDI----AAQIALKPAVNFHGGGHLNHTLFWENLAP--KSAGGGEPPSGALAKAI : 133
PficMnSOD1.       : -EEAKSKGNE----AAAASVAPLLNFHGGGHINHTLFWENLAPA-NQGGGGEPE-GALKKSI : 134
AlyrlMnSOD1.      : -DQAVNKGDA----STVVKLQSAIKFNGGGHVNHSIFWKNLAP--VNEGGGEPPKGSLGSAI : 129
NtabMnSOD1.       : -HNAISKGDA----PTVAKLHSAIKFNGGGHINHSIFWKNLAP--VREGGGEPPKGSLGWAI : 126
LpolMnSOD1.       : -AEAQAKSDT----SSIIALQPALKFNGGGHINHSIFWQNLSP----NGGGEPD-GDLMQLI : 125
BbeMnSOD1.        : -AEAQAKGDV----TTEIALGPALKFNGGGHLNHSIFWTNLSP----NGGGEPQ-GEVLEAI : 122
SkowMnSOD1.       : -KEATNKGDI----SKIISLAPALKFNGGGHLNHSIFWKNLSP----NGGGQPS-GDLLEQI : 122
HsMnSOD1.         : -QEALAKGDV----TAQTALQPALKFNGGGHINHSIFWTNLSP----NGGGEPK-GELLEAI : 121
Consensus         :                      p        h Nh  55  6 p         p    6   6
```

Suppl. Figure 3. Multiple sequence alignment showing essential regions of FeMnSOD obtained with Muscle implemented in MEGA X package [14]. Highly conserved regions responsible for metal binding are shown above the alignment. Color scheme: blue - highly conserved positions (above 95% of all aligned sequences), green - moderately conserved (between 75-95%), yellow - weakly conserved positions (between 50-75%). Numbers on the right represent corresponding amino acid position of each sequence.

```
                               ↓   ↓   ↓↓
                               b   b   bb                                  pos
CthedisFeSOD1. : FAPGGTDVQPLLCINTWEHVWLWDYGFGAGG-QGGKLAYAEAWWN-AINWEMVANEANIKRL : 302
CtheFeSOD1.    : FAPGGTDVQPVLCINTWEHVWLWDYGFGAGG-QGGKLAYAEAWWN-AINWEMVANEANIKRH : 302
TtheFeSOD.     : HAPGGTDLLPVLCINTWEHVWLWDYGFGVGAPDRGKLGYAEKWWK-HINWDLVLKEANVQRL : 301
TterFeSOD1.    : FAPGGTDLVPVLCLNTWEHVWLWDYGFGAGQAGGGKLEYAERWWN-LIDWERVQKEADIQRL : 305
CgloFeSOD1.    : HAPGGTDLVPVLCINTWEHVWLWDYGFGVGAPDRGKLGYAEKWWK-HVDWELVQKEADLQRL : 309
CgloFeSOD2.    : ---GG--DVPIFGVDMWEHAYYLQY-------LNGKAAYVKNIWN-VINWQTAQ-----ERF : 234
CcochFeSOD1.   : ---GG--DVAIFGVDMWEHAYYLQY-------LNGKAAYVENIWN-VINWQTAQ-----ERF : 232
PinfFeSOD.     : ---VDQPLIPILGLDVWEHAYYLKY-------QNRRPEYISAFWN-VANWDKVV-----EYY : 201
DeleFeSOD.     : ---QG---KVLLGLDVWEHAYYLDF-------RNRRPDYITNYLDKLANYEFAE-----AQL : 199
DponFeSOD1.    : ---HG--KIAVLTIDVWEHAYYIDF-------RNARPKYISTFLESLVNWDYAN-----AKY : 199
PequFeSOD.     : ---FG--DFPIISLDMWEHAYYLDY-------KEDRRRYVSNYMNHLVSWHSAT--LRIVRA : 271
ZaetFeSOD.     : ---WD--YNPILVVDVWEHAYYLDY-------QNRRPDFVSTFMDKLISWEAAS-----ARL : 256
CthedisMnSOD1. : ---TG-QLVPLMGIDAWEHAYYLQY-------ENRKAEYFEAIWN-VINWKTVA-----QRF : 224
CtheMnSOD1.    : ---TG-QLVPLMGIDAWEHAYYLQY-------ENRKAEYFEAIWN-VINWKTVA-----QRF : 224
TterMnSOD1.    : ---TG-PYVPLLGIDAWEHAYYLQY-------QNRKAEYFEAIWN-VINWKTVA-----ARF : 230
CcochMnSOD1.   : ---SG-PYVPLMGIDAWEHAYYLQY-------ENRKAEYFDAIWN-VINWKTVA-----SRF : 230
PexpMnSOD1.    : ---VG-QFEPLLGIDAWEHAYYLQY-------QNRKVEYFSAIWD-VINWKAAE-----KRF : 227
PficMnSOD1.    : ---TG-NYVPLLGIDAWEHAYYLQY-------QNRKAEYFDAIWN-VINWKTVA-----NRL : 228
AlyrlMnSOD1.   : ---GG-SLVPLVGIDVWEHAYYLQY-------KNVRPEYLKNVWK-VINWKYAS-----EVY : 226
NtabMnSOD1.    : ---KGANLVPLLGIDVWEHAYYLQY-------KNVRPDYLKNIWK-VMNWKYAN-----EVY : 223
LpolMnSOD1.    : ---TG--LIPLFGIDVWEHAYYLQY-------KNVRPDYVKAVWN-VVNWKDVS-----ERL : 221
BbeMnSOD1.     : ---TG--LVPLLGIDVWEHAYYLQY-------KNVRPDYVNAIWN-VVNWENIN-----ERF : 218
SkowMnSOD1.    : ---TG--LVPLLGIDVWEHAYYLQY-------KNVRPDYVKAIWE-IVNWKDVS-----ERF : 218
HsMnSOD1.      : ---TG--LIPLLGIDVWEHAYYLQY-------KNVRPDYLKAIWN-VINWENVT-----ERY : 217
Consensus:           g    p6  61 WEHa5y  5        n 4  5    w     5
```

Suppl. Figure 3 continued.
