# Supplementary material for: Parallel Molecular Evolution of Catalases and Superoxide Dismutases—Focus on Thermophilic Fungal Genomes
Source: Antioxidants (Basel). 2020 Oct 27;9(11):1047. doi: 10.3390/antiox9111047 (PMC7712995; doi:10.3390/antiox9111047)
Supplement: Supplementary file 1 [file antioxidants-09-01047-s001.zip › Supplementary Figure 4 (catalases_A distal_B proximal).pdf]

A)

```
                     ↓                                      ↓ ↓
                     d                                      d c            pos
CtheKat1.      : PERVVHARGSGAFGKFKLLEPIPELTMAGVLTDTSRETPVFVRFSTVLGSRGSADTVRDVRGFAT : 189
CtheKat2.      : PERAVHARGAGAHGTFTSYADWSNITAASFLNAAGKQTPVFVRFSTVAGSRGSADTVRDVHGFAI : 164
CtheKat3.      : LERMVHPCGSGAFGYFECTKDVTALTKANFLGTVGEKTPVFIRFSTVTFGREYPDQGRNPRGFAI : 172
CthedisKat2.   : PERAVHARGAGAHGTFTSYADWSNITAASFLNAAGKQTPVFVRFSTVAGSRGSADTARDVHGFAI : 164
CthedisKat3.   : LERMVHPCGSGAFGYFECTKDVTALTKANFLGTVGEKTPVFIRFSTVTFGREFPDQGRNPRGFAI : 172
MthKat1.       : PERVVHARGSGAFGKFRVFESAEDLTFAPVLTDTSRETPVFVRFSTVVGSRGSADTVRDVRGFAV : 151
MthKat2.       : PERAVHARGAGAHGTFTSYADWSNLTAASFLSSAGKKTPVFVRFSTVAGSRGSADTARDVHGFAT : 160
TterKat1.      : PERVVHARGSGAFGKFKVFESIEDLTFAPVLTDTTRETPVFVRFSTVLGSRGSADTVRDVRGFAV : 151
MythKat1.      : PERAVHARGAGAHGTFTSYADWSNITAASFLNATGKQTPVFVRFSTVAGSRGSADTARDVHGFAT : 160
MythKat4.      : PERAVHARGAGAHGTFTSYADWSNITAASFLNATGKQTPVFVRFSTTAGSRGSADTARDVHGFAT : 142
MamyKat1.      : PERVVHARGTGAFGKFRVFESAEDVTFAPVLTDTSRETPVFVRFSTVLGSRGSADTVRDVRGFAV : 151
MamyKat2.      : PERAVHARGAGAHGTFTSYSDWSNLTAASFLAAEGKETPVFIRFSTVAGSRGSADTVRDVHGFAT : 161
PanKat1.       : PERVVHARGSGAFGKFKLFESAEDVTFAPILTDTSRETPIFIRFSTVLGSRGSADTVRDVRGFAI : 150
PanKat2.       : PERAVHARGAGAHGTFTSYADWSNITAASFLGGAGKKTPVFVRFSTVAGSRGSADTARDVHGFAT : 164
SceKatA.       : PQRNPHAHGSGAFGYFEVTDDITDICGSAMFSKIGKRTKCLTRFSTVGGDKGSADTVRDPRGFAT : 129
SceKatT.       : PERVVHAKGGGCRLEFELTDSLSDITYAAPYQNVGYKCPGLVRFSTVGGESGTPDTARDPRGVSF : 134
NcKat1.        : PERVVHARGSGAFGKFKVYESASDLTMAPVLTDTSRETPVFVRFSTVLGSRGSADTVRDVRGFAV : 151
NcKat3.        : PERVVHARGAGAHGIFTSYGDWSNITAASFLGAKDKQTPVFVRFSTVAGSRGSADTARDVHGFAT : 161
NcKat4.        : PERVVHAKAAGAWGEFECTEDVSDFTSVDFLNKVGKKTKVLARISTVAGEKGSADTVRDIRGFAV : 121
AtKat1.        : PERVVHARGASAKGFFEVTHDITQLTSADFLRGPGVQTPVIVRFSTVIHERGSPETLRDPRGFAV : 124
AtKat2.        : PERVVHARGASAKGFFEVTHDISNLTCADFLRAPGVQTPVIVRFSTVIHARGSPETLRDPRGFAV : 124
AtKat3.        : PERVVHARGISAKGFFEVTHDISNLTCADFLRAPGVQTPVIVRFSTVVHGRASPETMRDIRGFAV : 124
PtroKat1.      : PERVVHAKGAGAFGYFEVTHDITKYSKAKVFEHIGKKTPIAVRFSTVAGESGSADTVRDPRGFAV : 134
HsKat1.        : PERVVHAKGAGAFGYFEVTHDITKYSKAKVFEHIGKKTPIAVRFSTVAGESGSADTVRDPRGFAV : 134
Consensus      : p2R VHa G gA G F         3 a  l      Tp   RFSTV g g adt R1  GFA
```

Suppl. Figure 4. Multiple sequence alignment showing conserved essential regions of typical heme catalases obtained with Muscle implemented in MEGA X package [14]. Presented highly conserved regions are A) distal side of heme & B) proximal side of heme. Color scheme: blue - highly conserved positions (above 95% of all aligned sequences), green - moderately conserved (between 75-95%), yellow - weakly conserved positions (between 50-75%). Numbers on the right represent corresponding amino acid position of each sequence.

A) (continued)

```
                                   ↓       ↓↓
                                   d       cc                                                    pos
CtheKat1.     : KFYTKEGNWDIVGNNIPVFFIQDAIKFPDVIHAGKPEPHNEVPQAQSAHNNFW---DFQFNHTE- : 250
CtheKat2.     : RFYTDEGNFDIVGNNIPVFFIQDAIRFPDLIHAVKPSPDSEIPQAATAHDSAW---DFFSSQPS- : 225
CtheKat3.     : KFYTLEGNYDI-----PIFFCRDPLQGPDVIRSQYRNPRNFLLDW----DACF---DLLANAPE- : 224
CthedisKat2.  : RFYTDEGNFDIVGNNIPVFFIQDAIRFPDLVHAVKPSPDSEIPQAATAHDSAW---DFFSSQPS- : 225
CthedisKat3.  : KFYTLEGNYDIVGLNFPIFFCRDPLQGPDVIRSQYRNPRNFLLDW----DACF---DLLANTPE- : 229
MthKat1.      : KFYTDEGNWDIVGNNIPVFFIQDAIKFPDVIHAVKPEPHNEVPQAQTAHNNFW---DFEFNHTE- : 212
MthKat2.      : RFYTDEGNFDIVGNNIPVFFIQDAIQFPDLIHAVKPRPDNEIPQAATAHDSAW---DFFSSQPS- : 221
TterKat1.     : KFYTQEGNWDIIGNDIPVFFIQDAIKFPDVIHAGKPEPHNEVPQAQSAHNNFW---DFQFNHTE- : 212
MythKat1.     : RFYTDEGNFDIVGNNIPVFFIQDAIQFPDLIHSVKPRPDNEIPQAATAHDSAW---DFFSQQPS- : 221
MythKat4.     : RFYTDEGNFDIVGNNIPVFFIQDAIQFPDLIHSVKPRPDNEIPQAATAHDSAW---DFFSQQPS- : 203
MamyKat1.     : KFYTQEGNWDIVGNNIPVFFIQDAIKFPDVIHAGKPEPHNEVPQAQSAHNNFW---DFQYNHSE- : 212
MamyKat2.     : RFYTDEGNFDIVGNNIPVFFIQDAIHFPDLIHAVKPRPDNEIPQAASAHDSAW---DFFSSQPS- : 222
PanKat1.      : KFYTQEGNWDIVANNIPVFFIQDAIKFPDVIHAGKPEPHNEVPQAQTAHNNFW---DFQYNHTE- : 211
PanKat2.      : RFYTDEGNFDIVGNNIPVFFIQDAIRFPDLIHSVKPSPDNEVPQAATAHDSAW---DFFSQQPS- : 225
SceKatA.      : KFYTEEGNLDWVYNNTPVFFIRDPSKFPHFIHTQKRNPQTNLRDA----DMFW---DFLT-TPEN : 186
SceKatT.      : KFYTEWGNHDWVFNNTPVFFLRDAIKFPVFIHSQKRDPQSHLNQFQDT-TIYW---DYLTLNPE- : 194
NcKat1.       : KFYTEEGNWDLVGNNIPVFFIQDAIKFPDVIHAGKPEPHNEVPQAQSAHNNFW---DFQFNHTE- : 212
NcKat3.       : RFYTDEGNFDIVGNNIPVFFIQDAIRFPDLIHSVKPSPDNEVPQAATAHDSAW---DFFSSQPS- : 222
NcKat4.       : KFFTEEGNWDFVGNDLPVFFIRDPVKFPSLNRSHKRHPQTNVPDS----TMFW---DFHNNNQE- : 178
AtKat1.       : KFYTREGNFDLVGNNFPVFFVRDGMKFPDMVHALKPNPKSHIQE-------NWRILDFFSHHPE- : 181
AtKat2.       : KFYTREGNFDLVGNNFPVFFIRDGMKFPDIVHALKPNPKSHIQE-------NWRILDFFSHHPE- : 181
AtKat3.       : KFYTREGNFDLVGNNTPVFFIRDGIQFPDVVHALKPNRKTNIQE-------YWRILDYMSHLPE- : 181
PtroKat1.     : KFYTEDGNWDLVGNNTPIFFIRDPILFPSFIHSQKRNPQTHLKDP----DMVW---DFWSLRPE- : 191
HsKat1.       : KFYTEDGNWDLVGNNTPIFFIRDPILFPSFIHSQKRNPQTHLKDP----DMVW---DFWSLRPE- : 191
Consensus     : 4F5T eGN D 6gn1 P6FF  D 6 fPd 6h  k  P   6          5   D
```

Suppl. Figure 4 continued.

B)

```
                                                       ↓   ↓
                                                       p   p             pos
CtheKat1.      : ELNRNPDEFFTQVEQIAFCTSH-VVPGIGFSDDPLLQGRNFSYFDTQISRL--GVNFQELPINRP : 437
CtheKat2.      : TLNRNPTNYFAETEQVMFQPGH-VVRGIDFSEDPLLQGRLFSYLDTQLNRH-GGPNFEQLPINMP : 413
CtheKat3.      : VLNKNPENFLRDVEQAAFSPGS-MVPGIEDSPDPLLQFRMFFYRDAQYHRL--GINLHQIPVN-- : 411
CthedisKat2.   : TLDRNPINYFAETEQVMFQPGH-VVRGIDFSEDPLLQGRLFSYLDTQLNRH-GGPNFEQLPINMP : 413
CthedisKat3.   : VLNKNPENFLRDVEQAAFSPGS-MVPGIEDSPDPLLQFRMFFYRDAQYHRL--GINLHQIPVN-- : 416
MthKat1.       : ELNRNPDEFFTQVEQIAFCTSH-IVPGIGFSDDPLLQGRNFSYFDTQISRL--GINFQELPINRP : 399
MthKat2.       : KLDRNPTNYFAETEQIMFQPGH-VVRGIDFSEDPLLQGRLFSYLDTQLNRN-GGPNFEQLPINMP : 409
TterKat1.      : ELNCNPDEFFTQVEQAAFCTSH-VVPGIGFSDDPLLQGRNFSYFDTQISRL--GVNFQELPVNRP : 399
MythKat1.      : KLDRNPTNYFAETEQVMFQPGH-IVRGIDFTEDPLLQGRLFSYLDTQLNRN-GGPNFEQLPINMP : 409
MythKat4.      : KLDRNPTNYFAETEQVMFQPGH-IVRGIDFTEDPLLQGRLFSYLDTQLNRN-GGPNFEQLPINMP : 391
MamyKat1.      : ELNRNPDEFFPQTEQIAFCTSH-VVPGIGFSDDPLLQGRNFSYFDTQISRL--GVNFQELPINRP : 399
MamyKat2.      : KLDRNPTNYFAETEQAMFQPGH-VVRGIDFSEDPLLQGRLFSYLDTQLNRH-GGPNFEQIPINMP : 410
PanKat1.       : ELNRNPDEFFPQTEQVAFCTSH-IVPGIGFSDDPLLQGRNFSYFDTQLSRL--GINWQELPINRP : 398
PanKat2.       : KLDRNPTNYFAETEQVMYQPGH-IVRGVDFTEDPLLQGRLFSYLDTQLNRNQGGPNFEQIPINRP : 414
SceKatA.       : VLNENPLNFFAQVEQAAFAPST-TVPYQEASADPVLQARLFSYADAHRYRL--GPNFHQIPVN-- : 373
SceKatT.       : TLTENVDNYFQEIEQVAFSPTNTCIPGIKPSNDSVLQARLFSYPDTQRHRL--GANYQQLPVNRP : 382
NcKat1.        : ELNRNPDEFFPQTEQIAFCTSH-VVNGIGFSDDPLLQGRNFSYFDTQISRL--GVNFQELPINRP : 399
NcKat3.        : TLNRNPMNYFAETEQISFQPGH-IVRGVDFTEDPLLQGRLYSYLDTQLNRH-RGPNFEQLPINRP : 410
NcKat4.        : RLNKNPENHFQDIEQAAFSPST-MVPGIGPSADPMLQARMFSYPDAARYRV--GPNYQQLP---- : 362
AtKat1.        : VLNKNIDNFFNENEQIAFCPAL-VVPGIHYSDDKLLQTRIFSYADSQRHRL--GPNYLQLPVNAP : 368
AtKat2.        : VLNKNIDNFFAENEQLAFCPAI-IVPGIHYSDDKLLQTRVFSYADTQRHRL--GPNYLQLPVNAP : 368
AtKat3.        : VLNRTIDNFFNETEQLAFNPGL-VVPGIYYSDDKLLQCRIFAYGDTQRHRL--GPNYLQLPVNAP : 368
PtroKat1.      : VLNRNPVNYFAEVEQIAFDPSN-MPPGIEASPDKMLQGRLFAYPDTHRHRL--GPNYLHIPVN-- : 376
HsKat1.        : VLNRNPVNYFAEVEQIAFDPSN-MPPGIEASPDKMLQGRLFAYPDTHRHRL--GPNYLHIPVN-- : 376
Consensus         L1 Np n5f   EQ  5 p      G6  3 Dp6LQ R 5sY D q  R   G N   6P6N p
```

Suppl. Figure 4 continued.
