# Supplementary material for: Parallel Molecular Evolution of Catalases and Superoxide Dismutases—Focus on Thermophilic Fungal Genomes
Source: Antioxidants (Basel). 2020 Oct 27;9(11):1047. doi: 10.3390/antiox9111047 (PMC7712995; doi:10.3390/antiox9111047)
Supplement: Supplementary file 1 [file antioxidants-09-01047-s001.zip › Supplementary Table 1.docx]

Supplementary Table 1. List of primers used for the detection and confirmation of transcription of *C. thermophilum* var*. dissitum* genes.

| Gene | Primer description | Sequence in 5´$\boldsymbol{\to}$ 3´ direction | Tm [° C] | PCR /RT-PCR  product size [bp] |
| --- | --- | --- | --- | --- |
| CuZnSOD1 | CuZnSOD1FULLfw | TTTCAGCTCTTATTTTCGCCTGC | 60.9 | g1105 /c674 |
|  | CuZnSOD1FULLrev | CACTAGCTGGATGTAATGGGC | 61.2 |  |
| CuZnSOD2 | CuZnSOD2FULLfw | AACAGCCTAATTCAACACACACC | 60.9 | g1164 /c1016 |
|  | CuZnSOD2FULLrev | AATGAACCGTCCGATAAAGATCC | 60.9 |  |
| FeSOD | FeSODFULLfw | GAGTATAGGGAAGTACGGGAAGG | 64.6 | g1183 /c1115 |
|  | FeSODFULLrev | GAGAGATCTACACAACTGGAACG | 62.9 |  |
| MnSOD1 | MnSOD1FULLfw | CTCACCCACATTACTATCTACGC | 62.9 | g959 /c769 |
|  | MnSOD1FULLrev | CAGGGATTACTTGGACACAGC | 61.2 |  |
| MnSOD2 | MnSOD2FULLfw | TGAATGTTCTTGTTGAGTTACCG | 59.2 | g949 /c890 |
|  | MnSOD2FULLrev | GAAGAGGATGATAGTTCTGAGGG | 62.9 |  |
